# Preventive Vitamin A Supplementation Improves Striatal Function in 6-Hydroxydopamine Hemiparkinsonian Rats

**DOI:** 10.3389/fnut.2022.811843

**Published:** 2022-02-01

**Authors:** Anaïs Marie, Julien Leroy, Morgane Darricau, Serge Alfos, Veronique De Smedt-Peyrusse, Emmanuel Richard, Sylvie Vancassel, Clementine Bosch-Bouju

**Affiliations:** ^1^Institut National de Recherche pour l'Agriculture, l'Alimentation et l'Environnement (INRAE), Institut Polytechnique de Bordeaux (INP), NutriNeuro, University of Bordeaux, Bordeaux, France; ^2^Institut des Maladies Neurodégénératives, University of Bordeaux, Bordeaux, France; ^3^Institut National de la Santé et de la Recherche Médicale (INSERM), Centre Hospital-Universitaire (CHU) Bordeaux, University of Bordeaux, Bordeaux, France

**Keywords:** vitamin A, dopamine, Parkinson's disease, 6-hydroxydopamine (6-OHDA), aldehyde dehydrogenase ALDH1A1, substantia nigra *pars compacta* (SNc), striatum

## Abstract

**Background:**

The mechanisms leading to a loss of dopaminergic (DA) neurons from the substantia nigra *pars compacta* (SNc) in Parkinson's disease (PD) have multifactorial origins. In this context, nutrition is currently investigated as a modifiable environmental factor for the prevention of PD. In particular, initial studies revealed the deleterious consequences of vitamin A signaling failure on dopamine-related motor behaviors. However, the potential of vitamin A supplementation itself to prevent neurodegeneration has not been established yet.

**Objective:**

The hypothesis tested in this study is that preventive vitamin A supplementation can protect DA neurons in a rat model of PD.

**Methods:**

The impact of a 5-week preventive supplementation with vitamin A (20 IU/g of diet) was measured on motor and neurobiological alterations induced by 6-hydroxydopamine (6-OHDA) unilateral injections in the striatum of rats. Rotarod, step test and cylinder tests were performed up to 3 weeks after the lesion. Post-mortem analyses (retinol and monoamines dosages, western blots, immunofluorescence) were performed to investigate neurobiological processes.

**Results:**

Vitamin A supplementation improved voluntary movements in the cylinder test. In 6-OHDA lesioned rats, a marked decrease of dopamine levels in striatum homogenates was measured. Tyrosine hydroxylase labeling in the SNc and in the striatum was significantly decreased by 6-OHDA injection, without effect of vitamin A. By contrast, vitamin A supplementation increased striatal expression of D2 and RXR receptors in the striatum of 6-OHDA lesioned rats.

**Conclusions:**

Vitamin A supplementation partially alleviates motor alterations and improved striatal function, revealing a possible beneficial preventive approach for PD.

## Introduction

Parkinson's disease (PD) is a neurodegenerative disease with multifactorial origins that involves interactions between genetic and environmental factors (1, 2). PD mainly results from the degeneration of dopamine (DA) neurons from the substantia nigra *pars compacta* (SNc) innervating the striatum (3). PD is characterized by disabling motor symptoms, such as akinesia, bradykinesia and tremor at rest, but these motor symptoms generally appear when a large proportion of DA neurons have already degenerated (4). In this context, nutrition has recently attracted attention as a potent modifiable environmental factor for the prevention of neurodegeneration in PD (5, 6).

Vitamin A (retinol) is a lipophilic vitamin that is critical for brain development and function along life (7). Vitamin A acts through its active metabolite, retinoic acid, that binds to nuclear receptors and modulate gene transcription (8). Supporting the relevance of vitamin A for PD, vitamin A-deficient rats display motor alterations (9) and conversely, DA neurons degeneration in mouse and rat PD models is prevented by pharmacological administration of retinoic acid or derivatives (10–13). Further supporting an implication of vitamin A signaling in PD is the observation that the sub-population of DA SNc neurons that is more prone to degenerate expresses the enzyme aldehyde dehydrogenase 1 subtype A1 (ALDH1A1) (8, 14–16), the synthesis enzyme of retinoic acid. Interestingly, mice lacking ALDH1A1 enzyme or ALDH1A1^+^ neurons, exhibit motor impairments along with alterations of DA metabolism (17, 18). This evidence highlights a role for vitamin A signaling in dopamine-related motor behaviors. However, the relevance of vitamin A nutritional supplementation for the prevention of PD, which can be easier to implement in humans compared to synthetic retinoids, has been overlooked.

Here, we hypothesized that preventive vitamin A supplementation can protect DA neurons, especially those expressing ALDH1A1 enzyme. We modeled DA fibers degeneration in rats with unilateral 6-hydroxydopamine (6-OHDA) lesion in the striatum (19). We investigated the impact of preventive vitamin A supplementation with dietary vitamin A (20 IU/g of diet) for 5 weeks before 6-OHDA or sham lesion. Motor behaviors were analyzed 1 to 3 weeks after the lesion. Post-mortem analyses were performed to study retinoid metabolism and DA transmission, with a focus on ALDH1A1^+^ DA neurons. This work shows that vitamin A supplementation in 6-OHDA rats improves striatal function, with a mild protective effect measured on ALDH1A1^+^ DA neurons.

## Materials and Methods

### Animals and Diet

Animals experiments were performed according to criteria of the European Communities Council Directive (2010/63/UE) and the French National Committee (authorization 16476-2018052314372190). All efforts were made to reduce the number of animals used and to minimize their suffering. A total of 66 male Wistar rats (Janvier Labs, France) was used (6-weeks old, 180–200g). Rats were housed 2 per cage and maintained in enriched and controlled environment (22°C ± 2°C, 40% of humidity), with a 12-h light/dark cycle (light on at 7 a.m.) with *ad libitum* access to water and food. Upon their arrival, rats were randomly allocated to two groups with different diet. One group received a diet with sufficient amount of retinol (5 IU retinol/g, INRAE, Jouy-en-Josas, 1 IU = 0.3 μg retinol), the second group received a similar diet but supplemented with vitamin A, with 20 IU retinol/g (INRAE, Jouy-en-Josas). The two diets differed only by the amount of retinol (retinyl acetate). Rats were weighted weekly and no body weight difference existed between the two groups at experiment onset. Animals were handled daily throughout the duration of the experiment.

### Surgery

After 5 weeks of dietary treatment (5 or 20 IU/g of diet), rats were subjected to unilateral stereotaxic injections in the striatum. Animals in both groups were randomly assigned to sham (0.9% saline containing 0.05 % of ascorbic acid, ref 95210 Sigma, St.Louis, MO, USA) or 6-hydroxydopamine (6-OHDA, 2 μg/μl in 0.9% saline and 0.05% of ascorbic acid solution, Sigma, St.Louis, MO, USA) injections. 6-OHDA is a neurotoxic dopaminergic analog carried out via monoamines transporters which allows the selective loss of catecholaminergic neurons, including dopaminergic and noradrenergic neurons (20). Unilateral injections in the striatum consisted in 2 injections of 3.5 μl each (7 μl/hemisphere in total). Briefly, Animals received carprofen (5 mg/kg, i.p, CARPROX VET®, Virbac, France) for analgesia and desipramine (15 mg/kg, i.p, ref D3900, Sigma, St.Louis, MO, USA) to protect noradrenergic neurons from 6-OHDA injection. Then, rats were placed on a stereotaxic apparatus (RWD life science) under body-temperature control and constant isoflurane anesthesia (3.5 % for induction, 1.5–2 % for maintenance, in 0.2% in air at 400 ml/min). The injected hemisphere (right or left striatum) was assigned randomly for each rat and the following coordinates for the 2 injection sites were chosen (19, 21): anteroposterior from bregma (AP) +1.2 and +0.2 mm; mediolateral (ML), ± 3.0–3.8 mm; dorsoventral (DV) −5 mm. Delivery rate for injections was 1.5 μl/min (10 μl 36G nanofil syringe, World Precision Instruments, USA, and ultra-micro pump UMP3, World Precision Instruments, USA) and the syringe was left in place for 5 min after each injection before slow removal. At the end of the injections, animals were sutured and lidocaine gel (Xylocaine ® 2 % Gel, AstraZeneca, UK) was applied on the sutures. Animals were kept on a heating pad until recovery and they were monitored twice a day for 4 days following the surgery.

### Behavioral Tests

#### Stepping Test

In order to quantify the akinesia of the contralateral forepaw induced by 6-OHDA injection, we performed stepping test as previously described (19, 22). For this test, animals were habituated for two consecutive days, three days before the first test. Briefly, rats were hold by a trained experimenter over a 90 cm-long bench and only one forepaw was placed on the bench. Rats were slowly moved along the bench (right to left and reverse) and the number of adjusted steps made by each paw on the bench was counted. The procedure was performed 3 times for each paw, on both directions. The stepping test was performed twice, the first test occurring before 6-OHDA (or sham) injection (named “pre-test” thereafter), and 3 weeks after for the second test. The result for each paw is the average of 3 consecutive trials. Results are presented as the percentage of steps made by the paw contralateral to the injected hemisphere (named “lesioned paw” thereafter) compared to the total number of steps. To assess the effect of 6-OHDA injection, the result of the test 3 weeks after the injection is normalized to pre-test.

#### Cylinder Test

Spontaneous forelimb use was tested in the cylinder test. In a clear plexiglass cylinder (diameter: 16 cm; height: 31 cm), we quantify the number of touches made by the rat with each forepaw on the cylinder wall while rearing during 3 min-sessions (23). The cylinder test was made at only one time point, 3 weeks after stereotaxic surgery. Results are presented as the percentage of touches made by the lesioned paw, compared to the total number of touches.

#### Rotarod Test

Motor coordination and balance of rats were tested with rotarod test (Rota-Rod ENV-578(R), MedAssociates) as previously described (24). Briefly, rats were placed on a rotative axis and their latency to fall was measured. Each session had a maximum duration of 5 min. Three days before the first test, training sessions were performed on three consecutive days, with two sessions per day at constant speed (day 1 at 12 rpm, day 2 at 10 rpm, day 3 morning at 12 rpm and day 3 afternoon 14 rpm). For test sessions, the rotative axis followed a linear acceleration from 5 to 45 rpm. Test sessions were performed 1 week before, and 1, 2 and 3 weeks after striatal unilateral injections. Data are presented as latency to fall (s) for each animal, as automatically registered by the apparatus.

### Sample Collection

Three weeks after stereotaxic injections, animals were euthanized following 2 different procedures. On one side, 36 rats were deeply anesthetized with isoflurane (5 %) and decapitated. Blood was collected from the trunk and brain was immediately frozen in isopentane and stored at −80°C. The liver was also collected and stored at −80°C for retinol assay. On the other side, 30 rats were euthanized with intraperitoneal injection of hexagon (150 mg/Kg)/lidocaine (230 mg/Kg). Blood was collected *via* an intracardiac puncture and then rats were transcardially perfused with paraformaldehyde (PFA) 4% solution in order to fix brain tissues. Brains were quickly extracted, post-fixed in PFA 4% for 24 h and plunged in 30 % sucrose solution for 48 h before storage at −80°C. All Blood samples were centrifuged (10 min, 10 000 x g, 4°C) and plasma was stored at −80°C until used.

### Retinol Assays

Retinol was measured in liver extracts (the storage organ of vitamin A), that have been homogenized in sodium phosphate-EDTA (0.05 M, pH 7.8) buffer, and in the plasma. Retinol was extracted with a solution of tocopherol acetate (1.057 mmol/L, T3376, Sigma St.Louis, MO, USA) used as internal standard, added with a solution of hexane and butyl-hydroxy-toluene (20 mg/mL, ref B1378, Sigma St.Louis, MO, USA). Homogenates were centrifuged (2 min, 20°C, 16,000 × *g*) and the supernatant containing retinol was recovered and evaporated under nitrogen. Following evaporation, residues were treated with methanol and assayed by high performance liquid chromatography (HPLC) as previously described (25). Two vitamin A calibration standard (Chromsystems 34004) were processed in parallel with samples. Results are given in μmol/g for the liver and in μmol/L for the plasma.

### Monoamines Measures

HPLC analyses were performed on 1 mm-punches of the striatum from brains frozen in isopentane and then stored at −80°C. To do so, 200 μm-coronal brain sections were performed using a cryostat tissue slicer (Leica Biosystems, Germany). According to rat brain atlas (21), punches of the striatum were taken between 2.20 and −1.20-mm from bregma. Punches were immediately stored at −80°C.

As previously described (26) monoamines were extracted from striatum punches using a TissuLyser system (Qiagen, Courtaboeuf, France) in extraction buffer containing 12 mM perchloric acid, 56 μM EDTA, 0.26 mM sodium disulfite and 3 mM octanesulfonic acid. One part of the supernatant (containing proteins) was frozen at −80°C for subsequent western-blot analysis, the other part (300 μL) was used for measure of monoamines content. Total protein level for each sample was quantified using bicinchoninic acid (BCA) assay, in order to normalize HPLC results.

The monoamines of interest, DA and its main metabolites, dihydroxyphenylacetic acid (DOPAC) and homovanillic acid (HVA) were identified using HPLC coupled with an electrochemical detector as previously described (26). Results for the lesioned side were normalized to the control side for each animal.

### Western Blot

Expression levels of proteins (RXR, D2R and ALDH1A1) were measured by western-blot in striatum punches. Briefly, 10 μg of proteins with 2 μL of NaOH 1 M were added to loading buffer to basify the extraction medium used for monoamines extraction. Proteins samples were loaded on 12 % sodium polyacrylamide-dodecyl sulfate gel and then transferred to nitrocellulose membranes. Membranes were saturated with a solution of PBS-tween (0.1%, TWEEN®20, P1379, Sigma St.Louis, MO, USA) milk (5%) and incubated overnight at 4°C with different primary antibodies (Table 1). After 1 h-incubation with secondary antibody solution (Table 1) coupled to horseradish peroxidase (HRP), chemiluminescence has been detected with peroxidase revealing solution (SuperSignal West Dura, ThermoFisher, Waltham, MA, USA) and were revealed using ChemiDoc MP (Biorad, Hercules, CA, USA). Signal intensity was quantified and normalized on GAPDH (Glyceraldehyde 3-phosphate dehydrogenase) or α-tubuline protein expression for each sample. Results are given in % of the lesioned side, normalized to the control side.

**Table 1:** Reagents and resources used for western blot experimentation.

	**Antibodies**	**Host species**	**Dilution**	**Reference**
Primary	ALDH1A1	Rabbit	1:2000	HPA002123, Sigma
Secondary	Anti-rabbit IgG-HRP conjugated	Donkey	1:5000	711-035-152, Jackson ImmunoResearch
Primary	RXRγ	Rabbit	1:1500	ab53162, Sigma
Secondary	Anti-rabbit IgG-HRP conjugated	Donkey	1:5000	711-035-152, Jackson ImmunoResearch
Primary	D2R	Rabbit	1:2000	AB5084P, Merck
Secondary	Anti-rabbit IgG-HRP conjugated	Donkey	1:5000	711-035-152, Jackson ImmunoResearch
Primary	GAPDH	Rabbit	1:15 000	D16H11, Cell Signaling
Secondary	Anti-rabbit IgG-HRP conjugated	Donkey	1:20 000	711-035-152, Jackson ImmunoResearch
Primary	α-tubuline	Mouse	1:10 000	T5168, Sigma
Secondary	Anti-mouse IgG-HRP conjugated	Donkey	1:10 000	715-035-151, Jackson ImmunoResearch

### Immunofluorescence Staining

Coronal sections of 40-μm thickness were made using a cryostat tissue slicer (Leica Biosystems, Germany) from brains post-fixed in PFA and stored at −80°C. According to (27), we selected 3 levels of interest for striatum and SNc. The 3 levels were designated as anterior (bregma striatum: +2.2, SNC: −5.2), intermediate (bregma striatum: +1.6, SNC: −5.3) and posterior (bregma striatum: +0.7, SNC: −5.8). Sections were processed for simple or double-fluorescent immunohistochemistry. After washes with PBS, sections were blocked with appropriate blocking solution depending on primary antibodies (Table 2) for 1 h at room temperature. Sections were then incubated with primary antibodies diluted in blocking solution, overnight at 4°C. The next day, sections were washed with blocking solution [or PBS for IBA1 (Ionized calcium binding adapter molecule 1) labeling] and incubated with the secondary antibody conjugated to a fluorochrome for 2 h at room temperature, protected from light. Sections were washed with PBS and then mounted in mounting medium with DAPI (sc-359850, CliniSciences, France).

**Table 2:** Reagents and resources used for immunostaining experimentation.

	**Antibodies**	**Host species**	**Dilution**	**Blocking solution**	**Reference**
Primary	TH	Mouse	1:1000	3% bovine serum albumine,	mab 318, Merck
Secondary	Anti-mouse IgG-Alexa 488	Donkey	1:1000	0.3% Triton X-100, PBS	ab150105, Abcam
Primary	ALDH1A1	Rabbit	1:1000	3% bovine serum albumine,	HPA002123, Sigma
Secondary	Anti-rabbit IgG-Alexa 568	Donkey	1:1000	0.3% Triton X-100, PBS	ab175470, Abcam
Primary	IBA1	Rabbit	1:500	10% normal donkey serum,	019-19741, Fujifilm
Secondary	Anti-rabbit IgG-Alexa 488	Goat	1:1000	0.5% Triton X-100, PBS	A11008, Invitrogen

### Image Analysis

All striatum sections were scanned using a widefield microscope (Hamamatsu Nanozoomer 2.0 HT) with 20X (20X, NA 0.75) objective to visualize the whole striatum. SNc sections were scanned using a laser scanning confocal microscope (Leica DM5500 TCS SPE) with 40X oil-immersion objective to visualize dopaminergic neurons in the tissue. Setting parameters for acquisition, such as laser power or photomultipliers gain, were kept constant between all animals for a given labeling. Digital images obtained were processed with Image J software. In order to limit analysis bias, we used semi-automated quantification with macros on Image J.

For the striatum, image of each brain section was divided in two parts, the lesioned side, corresponding to the injected hemisphere (with 6-OHDA or saline solution) and the control side. Each side has been analyzed. For TH and ALDH1A1 staining in the striatum, we quantified the intensity of staining. We assigned green color to TH-associated fluorescence channel and magenta color to ALDH1A1-associated fluorescence channel. From TH channel, we selected the striatum from the whole brain section using the “wand” set tool in Image J, which defines the region of interest (ROI). Area and staining intensity for TH and ALDH1A1 were measured based on this ROI.

For IBA1 labeling, only the lesioned side was analyzed. We assigned green color to IBA1-associated fluorescence and we first delimited the striatum using the “freehand” set tool in image J. Then we defined parameters for “subtract background” and “rolling” that we applied to all brain sections analyzed. These tools allowed us to better isolate activated microglia labeled by IBA1 and count them afterwards. To select each activated microglia, we used the tool “process find maxima” and “analyze particles” with common thresholds for all cuts. Finally, we measured the area of the striatum previously delimited and we used the function “count” to determine the number of IBA1 positive microglia in the striatum. Results are expressed in number of IBA1^+^/ striatum area (mm^2^) which correspond to a density of activated microglia. We also quantified the total intensity of staining for IBA1 in the striatum contralateral to the lesion, using the ROI previously defined for the counting.

For SNc, final images of the whole SNc correspond to a mosaic of several images taken individually at 40X. Both sides of SNc, lesioned and control, were imaged and analyzed. For TH and ALDH1A1 labeled in SNc, we quantified the number of positive neurons. Similar to the striatum, we assigned green color for TH staining and magenta color for ALDH1A1 staining. The silver color on figures corresponds to the merge of both colors and indicates TH and ALDH1A1 positive neurons. From TH channel, we isolated neurons from the background with a median filter and applied a threshold with common parameters for all cuts. Then, we selected labeled neurons with the tool “analyze particles” and we used the function “count” to determine the number of TH positive neurons in the SNc. The process was repeated for ALDH1A1 and merge channels, in order to determine the number of TH+ALDH1A1 positive neurons. Note that all ALDH1A1 positive neurons were also TH positive neurons.

These analyses were carried out for the anterior, intermediate and posterior level of striatum and SNc. For the striatum, results of the lesioned side are expressed as normalized to the staining intensity of the control side.

### Statistical Analysis

Data were analyzed using GraphPad prism 7.0 an 9.2.0 (Graphpad software). Two-way ANOVA were used when the effects of 2 factors (factors “vitamin A diet” and “6-OHDA injection”) were tested. Three-way ANOVA (mixed-effects model) were performed for repeated data across time points (factors “diet”, “6-OHDA” and “time”). In case of significant interaction for 2-way and 3-way ANOVAs (*p* ≤ 0.06), a *post-hoc* test was performed based on the two-stage step-up method of Benjamini. All data are presented as means ± SEM. Details of the statistical analysis are summarized in Table 3. Statistical significance in statistics tables is expressed as ^*^p < 0.05, ^**^p < 0.01, ^***^p < 0.001. The significance on the figure is expressed with letter a, b, c and d, which differed from each other. The notation ab indicates that the experimental group does not differ from either group with notation a or notation b.

**Table 3:** Summary of statistical analysis.

**Figure 2**	**Statistical test**	***n***	**Outcome measure**	**6-OHDA effect**		**Vitamin A effect**		**Time effect**		
Figure 2A: Body weight	3-way ANOVA	16–18	Body weight (g)	F_(1,62)_ = 0.2926	*p =* 0.591	F_(1,62)_ = 0,0802	*p =* 0.778	F_(1.494,92.65)_ = 3.778	*p* < 0.001^***^	
				**6-OHDA effect**		**Vitamin A effect**		**Interaction**		***post-hoc*** **test**
Figure 2B: liver retinol level	2-way ANOVA	7–9	Retinol level (μmol/g)	F_(1,29)_ = 0.6026	*p =* 0.443	F_(1,29)_ = 15,19	*p* < 0.001^***^	F_(1,29)_ = 0,03328	*p =* 0.856	
Figure 2C: Plasma retinol level	2-way ANOVA	8–9	Retinol level (μmol/l)	F_(1,31)_ = 4.661	*p =* 0.038^*^	F_(1,31)_ = 2,687	*p =* 0.111	F_(1,31)_ = 0,9726	*p =* 0.331	
Figure 2D: RXRγ expression	2-way ANOVA	5–8	RXRγ/GAPDH (AU)	F_(1,23)_ = 0.3524	*p =* 0.558	F_(1,23)_ = 7,447	*p =* 0.012^*^	F_(1,23)_ = 0,03963	*p =* 0.843	
Figure 3				**6-OHDA effect**		**Vitamin A effect**		**Interaction**		
Figure 3A: Step test - Pre-test	2-way ANOVA	16–18	Total # steps	F_(1,62)_ = 0.1438	*p =* 0.706	F_(1,62)_ = 0.0255	*p =* 0.874	F_(1,62)_ = 0.6536	*p =* 0.422	
Figure 3A: Step test	2-way ANOVA	16–18	step test score	F_(1,62)_ = 15.21	*p* < 0.001^***^	F_(1,62)_ = 0.5599	*p =* 0.457	F_(1,62)_ = 0.2593	*p =* 0.612	
Figure 3B: Cylinder test number total of steps	2-way ANOVA	16–18	Total # touches	F_(1,62)_ = 4.704	*p =* 0.033^*^	F_(1,62)_ = 0,05835	*p =* 0.809	F_(1,62)_ = 0,07746	*p =* 0.781	
Figure 3B: Cylinder test	2-way ANOVA	16–18	Touches (% ctrl paw)	F_(1,62)_ = 27.83	*p* < 0.001^***^	F_(1,62)_ = 0,8238	*p =* 0.367	F_(1,62)_ = 7,439	*p =* 0.008^**^	5 IU sham vs 5 IU 6-OHDA *p* < 0.001; 20 IU sham vs 5 IU 6-OHDA *p =* 0.006; 20 IU 6-OHDA vs 5 IU 6-OHDA *p =* 0.007
Figure 3C: Rotarod week−1	2-way ANOVA	16–18	Time on rotarod (s)	F_(1,62)_ = 0.1225	*p =* 0.727	F_(1,62)_ = 0,0441	*p =* 0.834	F_(1,62)_ = 0,04134	*p =* 0.839	
				**6-OHDA effect**		**Vitamin A effect**		**Time effect**		
Figure 3C: Rotarod	3-way ANOVA	16–18	Time on rotarod (s)	F_(1,62)_ = 3.687	*p =* 0.059^#^	F_(1,62)_ = 0.01228	*p =* 0.912	F _(1.938,120.2)_ = 1.860	*p =* 0.161	
Figure 4				**6-OHDA effect**		**Vitamin A effect**		**Interaction**		**PostHoc test**
Figure 4A: DA	2-way ANOVA	6–9	DA Level (% ctrl side)	F_(1,28)_ = 57.82	*p* < 0.001^***^	F_(1,28)_ = 0,141	*p =* 0.710	F_(1,28)_ = 0,5414	*p =* 0.468	
Figure 4B: DOPAC	2-way ANOVA	6–9	DOPAC Level (% ctrl side)	F_(1,28)_ = 17.9	*p* < 0.001^***^	F_(1,28)_ = 0,6695	*p =* 0.420	F_(1,28)_ = 0,03669	*p =* 0.849	
Figure 4C: HVA	2-way ANOVA	6–9	HVA Level (% ctrl side)	F_(1,28)_ = 30.74	*p* < 0.001^***^	F_(1,28)_ = 0,7256	*p =* 0.401	F_(1,28)_ = 0,4844	*p =* 0.492	
Figure 4D: DA/DOPAC	2-way ANOVA	7–9	Ratio	F_(1,29)_ = 7.079	*p =* 0.012^*^	F_(1,29)_ = 0,3332	*p =* 0.568	F_(1,29)_ = 1,024	*p =* 0.319	
Figure 4E: DA/HVA	2-way ANOVA	7–9	Ratio	F_(1,29)_ = 4.185	*p =* 0.050^*^	F_(1,29)_ = 0,2515	*p =* 0.619	F_(1,29)_ = 0,02674	*p =* 0.871	
Figure 5				**6-OHDA effect**		**Vitamin A effect**		**Interaction**		***post-hoc*** **test**
Figure 5B: TH intermediate striatum	2-way ANOVA	6–7	TH Intensity (%ctrl side)	F_(1,23)_ = 207.2	*p* < 0.001^***^	F_(1,23)_ = 0,02812	*p =* 0.868	F_(1,23)_ = 3,779	*p =* 0.064^#^	5 IU sham vs. 5 IU 6-OHDA *p* < 0.001; 20 IU sham vs. 20 IU 6-OHDA *p* < 0.001; 5 IU sham vs. 20 IU 6-OHDA *p* < 0.001; 20 IU sham vs 5 IU 6-OHDA *p* < 0.001
Figure 5C: TH posterior striatum	2-way ANOVA	5–8	TH Intensity (%ctrl side)	F_(1,22)_ = 210.5	*p* < 0.001^***^	F_(1,22)_ = 1,224	*p =* 0.280	F_(1,22)_ = 5,718	*p =* 0.025^*^	5 IU sham vs. 20 IU sham *p =* 0.031; 5 IU sham vs. 5 IU 6-OHDA *p* < 0.001; 20 IU sham vs. 20 IU 6-OHDA *p* < 0.001; 5 IU sham vs. 20 IU 6-OHDA *p* < 0.001; 20 IU sham vs. 5 IU 6-OHDA *p* < 0.001
Figure 5E: TH intermediate SNc	2-way ANOVA	6–9	# TH neurons	F_(1,25)_ = 17.66	*p* < 0.001^***^	F_(1,25)_ = 0,1169	*p =* 0.735	F_(1,25)_ = 0,2052	*p =* 0.654	
Figure 5F: TH posterior SNc	2-way ANOVA	6–8	# TH neurons	F_(1,24)_ = 5.407	*p =* 0.028^*^	F_(1,24)_ = 0,2853	*p =* 0.598	F_(1,24)_ = 0,5264	*p =* 0.475	
Figure 6	**Statistical test**	***n***	**Outcome measure**	**6-OHDA effect**		**Vitamin A effect**		**Interaction**		***post-hoc*** **test**
Figure 6: D2R expression	2-way ANOVA	8–9	D2R/α-tubuline	F_(1,29)_ = 8.001	*p =* 0.008^**^	F_(1,29)_ = 2,031	*p =* 0.164	F_(1,29)_ = 6,87	*p =* 0.013^*^	5 IU sham vs. 5 IU 6-OHDA *p* < 0.001; 20 IU sham vs. 5 IU 6-OHDA *p =* 0.006; 5 IU 6-OHDA vs. 20 IU 6-OHDA *p =* 0.007
**Figure 7**				**6-OHDA effect**		**Vitamin A effect**		**Interaction**		***post-hoc*** **test**
Figure 7B: ALDH1A1 intermediate SNc	2-way ANOVA	6–9	# ALDH1A1 neurons	F_(1,25)_ = 26.38	*p* < 0.001^***^	F_(1,25)_ = 3,186	*p =* 0.086^#^	F_(1,25)_ = 0,4866	*p =* 0.491	5 IU sham vs. 20 IU sham *p =* 0.09 #; 5 IU sham vs. 5 IU 6-OHDA *p =* 0.006; 20 IU sham vs. 20 IU 6-OHDA *p* < 0.001; 5 IU sham vs. 20 IU 6-OHDA *p =* 0.019; 20 IU sham vs. 5 IU 6-OHDA *p* < 0.001
Figure 7C: ALDH1A1 posterior SNc	2-way ANOVA	6–8	# ALDH1A1 neurons	F_(1,24)_ = 13.25	*p =* 0.001^**^	F_(1,24)_ = 1,248	*p =* 0.275	F_(1,24)_ = 0,7636	*p =* 0.390	
Figure 7E: Merge intermediate SNc	2-way ANOVA	5–9	% ALDH1A1 neurons	F_(1,24)_ = 5.675	*p =* 0.025^*^	F_(1,24)_ = 1,247	*p =* 0.275	F_(1,24)_ = 0,1778	*p =* 0.677	
Figure 7F: Merge posterior SNc	2-way ANOVA	6–8	% ALDH1A1 neurons	F_(1,24)_ = 11.09	*p =* 0.002^**^	F_(1,24)_ = 1,921	*p =* 0.178	F_(1,24)_ = 0,0028	*p =* 0.958	
Figure 8				**6-OHDA effect**		**Vitamin A effect**		**Interaction**		***post-hoc*** **test**
Figure 8A: ALDH1A1 expression	2-way ANOVA	8–9	ALDH1A1/ GAPDH (AU)	F_(1,29)_ = 19.28	*p* < 0.001^***^	F_(1,29)_ = 0,05594	*p =* 0.814	F_(1,29)_ = 0,3644	*p =* 0.550	
Figure 8C: ALDH1A1 intermediate striatum	2-way ANOVA	6–7	ALDH1A1 Intensity (AU)	F_(1,23)_ = 58.71	*p* < 0.001^***^	F_(1,23)_ = 0,6587	*p =* 0.425	F_(1,23)_ = 1,466	*p =* 0.238	
Figure 8D: ALDH1A1 posterior striatum	2-way ANOVA	5–8	ALDH1A1 Intensity (AU)	F_(1,22)_ = 87.44	*p* < 0.001^***^	F_(1,22)_ = 3,488	*p =* 0,075 ^#^	F_(1,22)_ = 2,146	*p =* 0.157	5 IU sham vs. 20 IU sham *p =* 0.039 5 IU sham vs. 5 IU 6-OHDA *p* < 0.001 20 IU sham vs. 20 IU 6-OHDA *p* < 0.001 5 IU sham vs. 20 IU 6-OHDA *p* < 0.001 20 IU sham vs 5 IU 6-OHDA *p* < 0.001

*Statistical significance was assessed as*

# *p < 0.09*,

* *p < 0.05*,

** *p < 0.01*,

*** *p < 0.001*.

## Results

### Impact of 6-OHDA Injection and Dietary Vitamin A on Retinol Levels

**Figure 1:**
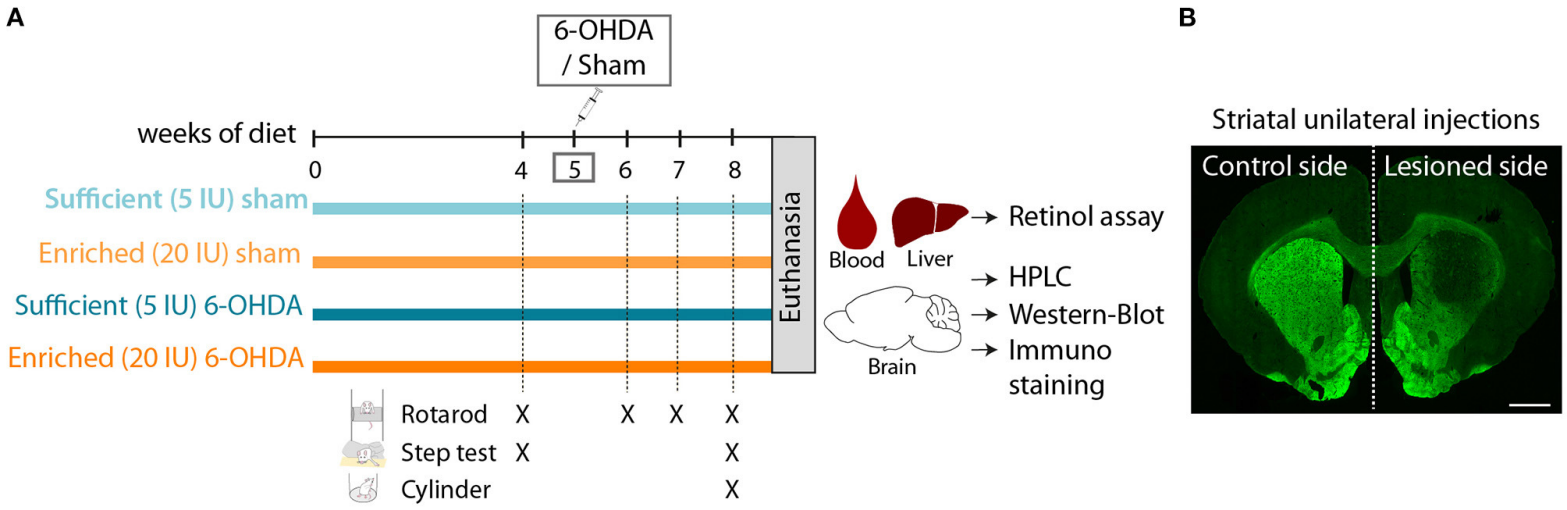
Experimental design. **(A)** Schematic overview of experimental design. 5 IU and 20 IU stand for 5 IU/g of diet and 20 IU /g of diet, respectively. **(B)** Representative image of rat striatum (bregma +1.6) injected with 6-OHDA (lesioned side), labeled with TH immunofluorescence (green). Scale bar: 1000 μm.

**Figure 2:**
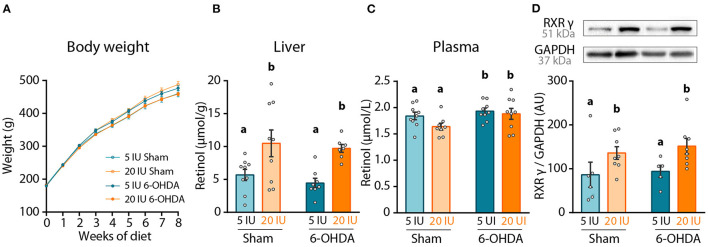
Impact of dietary vitamin A and 6-OHDA injection on retinol levels and striatum RXRγ expression. **(A)** Measure of rats' body weight (g) throughout experiment. **(B,C)** Levels of retinol in the liver (μmol/g) **(B)**, and in the plasma (μmol/L) **(C)**. **(D)** Representative western blot for retinoid X receptor gamma (RXRγ) in striatum homogenates (upper inset) and quantification (graph). Protein expression levels were normalized with GAPDH for each sample. Results are expressed as means ± SEM, with individual data points for histograms. a, b: values significantly different (*p* < 0.005). Details of the statistical analysis are summarized in Table 3.

Adult male rats were subjected to 5 weeks of dietary treatment, either sufficient diet (5 IU retinol/g of diet, referred to as 5 IU thereafter) or supplemented diet (20 IU retinol/g of diet, referred to as 20 IU thereafter). Rats then received a sham or 6-OHDA unilateral injection in the striatum, leading to 4 experimental groups (Figure 1). Dietary treatments were continued for 3 additional weeks during which, behavioral tests were performed. Body weight curves were not different between the 4 groups (6-OHDA effect *p* = 0.591; diet effect: *p* = 0.778), indicating that dietary treatment and intrastriatal injections had no effect on body weight (Figure 2A).

The effect of dietary treatment was first assessed on retinol levels, both in the plasma and the liver. Vitamin A supplemented diet almost doubled retinol levels in the liver (*p* < 0.001), but did not alter retinol levels in the plasma (*p* = 0.111) (Figures 2B,C). These results confirm the impact of dietary vitamin A treatment on retinol metabolism. Of note, 6-OHDA injection slightly but yet significantly increased retinol levels in the plasma (*p* = 0.038), but not in the liver (*p* = 0.443).

In the brain, the best proxy for retinoid signaling is the measure of retinoids receptors, since their expression is directly controlled by retinoic acid levels (28, 29). Thus, we quantified in the striatum the expression of retinoid X receptor (RXRγ), an isoform highly expressed in this structure (30). Vitamin A enriched diet significantly increased RXRγ expression in both sham and 6-OHDA-injected rats (*p* = 0.012) (Figure 2D). Of note, 6-OHDA injection did not modify expression of RXRγ in the striatum (*p* = 0.558).

Additionally, we quantified microglia in the striatum, as reflected by IBA1 staining, since 6- OHDA and dietary vitamin A can act on neuro-inflammatory processes (8, 31). In our conditions, quantification of the number of IBA1^+^ cells, as well as total IBA1 intensity in the striatum did not reveal any significant effect of 6-OHDA or vitamin A supplementation (Supplementary Figure 1).

Altogether these results validate the positive impact of 8-week dietary vitamin A supplementation on retinol function at the periphery and in the striatum.

### Impact of 6-OHDA Injection and Dietary Vitamin A on Motor Behavior

The effect of preventive vitamin A supplementation and 6-OHDA injection on motor behavior was measured with three classical tests: the stepping test, the cylinder test and the rotarod test.

**Figure 3:**
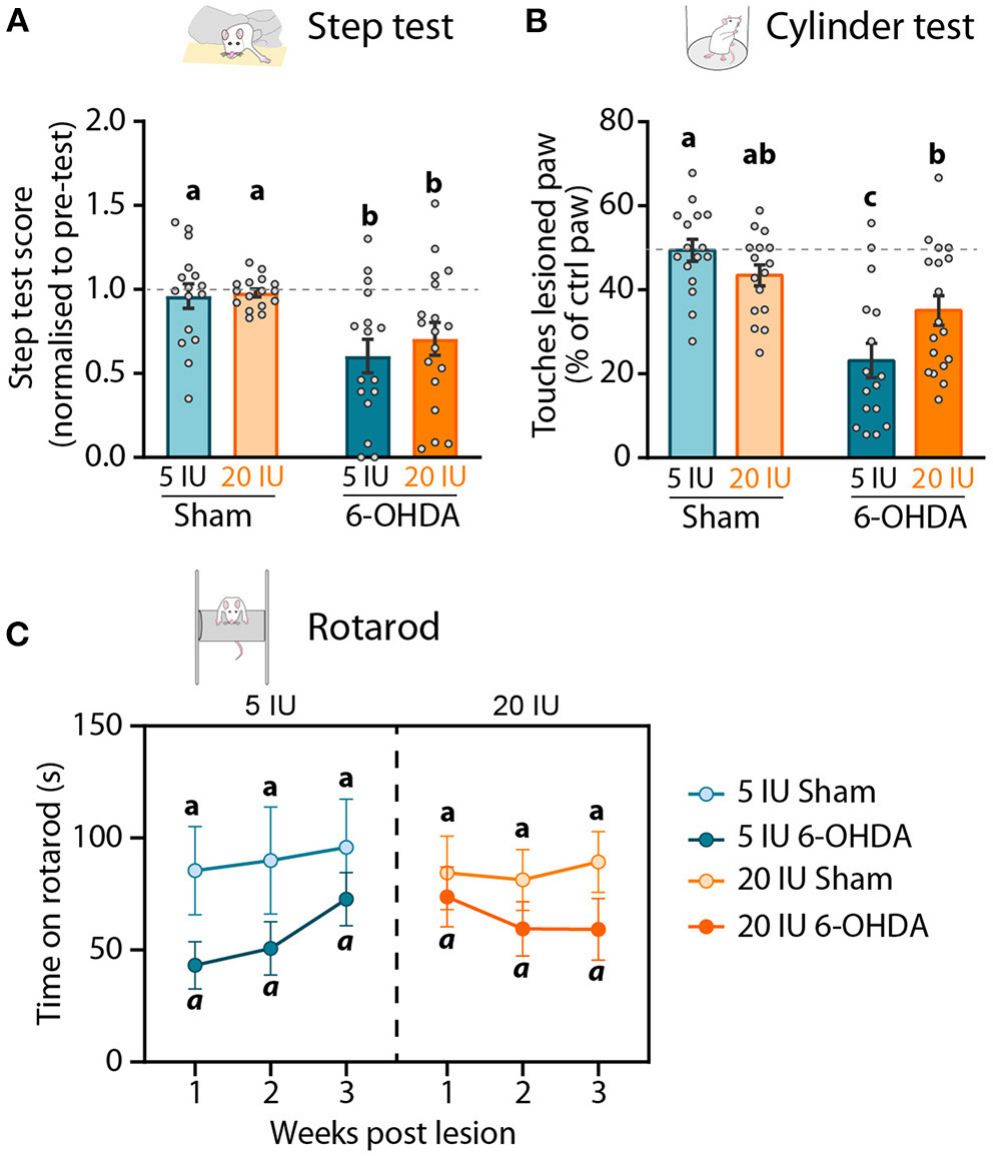
Impact of 6-OHDA injection and dietary vitamin A on motor behavior. **(A)** Step test: Step test score 3 weeks after the lesion represents the number of steps made by the lesioned paw as the percent (%) of the total number of steps, normalized to the step test performed before the lesion. **(B)** Cylinder test: number of touches made by the lesioned paw expressed as % of total touches made by the control paw the cylinder test 3 weeks after the lesion. **(C)** Rotarod test: latency to fall (s) of rats from the apparatus 1, 2 and 3 weeks after the lesion. Results are expressed as means ± SEM, with individual data points for histograms. a–c: values significantly different (*p* < 0.005, *a*: *p* = 0.059). Details of the statistical analysis are summarized in Table 3.

The stepping test was used to validate the impact of unilateral injection of 6-OHDA on the mobility of the paw contralateral to the lesion. The stepping test training and the first stepping session were performed before the unilateral injection, and the number of steps made by both paws was not different between groups (Table 3). Three weeks after 6-OHDA injection, the number of steps made by the paw contralateral to the lesion (named ‘lesioned paw’ thereafter) was reduced by half compared to sham rats (6-OHDA effect: *p* < 0.001) (Figure 3A). No significant effect of vitamin A supplementation was observed.

In the cylinder test, rats were freely moving and the number of touches made by each paw on the wall of the cylinder while rearing was quantified. This test was used to measure forelimb akinesia. In order to avoid habituation, this test was performed only once, 3 weeks after the lesion. In total, sham rats made significantly more paw touches than 6-OHDA lesioned rats (6-OHDA effect: *p* = 0.033). Under sufficient diet, 6-OHDA rats used significantly less their lesioned paw, compared to sham rats (5 IU sham vs. 5 IU 6-OHDA: *p* < 0.001). By contrast, the use of the lesion paw in vitamin A supplemented rats was not significantly different between sham and 6-OHDA rats (20 IU sham vs. 20 IU 6-OHDA: *p* = 0.072) (Figure 3B). Proportion of touches made with the lesioned paw was significantly higher for 6-OHDA rats under vitamin A supplementation, compared to 6-OHDA rats without supplementation (5 IU 6-OHDA vs. 20 IU 6 OHDA: *p* = 0.007).

In the rotarod test, we measured the latency to fall of rats placed on an accelerating rotative axis (4–40 rpm). The rotarod training and the first test were performed before the lesion, in order to ensure the absence of initial difference between groups (Table 3). Rotarod tests were then performed 1, 2 and 3 weeks after the lesion. In sham rats under sufficient or enriched diet, latency to fall remained stable across sessions (Figure 3C). In 6-OHDA rats, the 3-way ANOVA analysis revealed a strong tendency for shorter latency to fall, compared to sham rats (*p* = 0.059). Despite statistical analysis did not reveal significant effect, we observed that the impact of the lesion was not stable across time, and differ between 6-OHDA rats under sufficient or supplemented diet. In 6-OHDA rats under sufficient diet, latency to fall was more affected 1 week after the lesion that 3 weeks later. Conversely, in 6-OHDA rats with supplemented diet, latency to fall was close to sham rats 1 week after the lesion, but was shorter at 2 and 3 weeks after the lesion.

Taken together, these results reveal that beneficial effect of vitamin A supplementation on motor impairments induced by unilateral 6-OHDA injection in the striatum is only observable in the cylinder test.

### Impact of 6-OHDA Lesion and Dietary Vitamin A on Dopamine Transmission in the Nigro-Striatal Pathway

**Figure 4:**
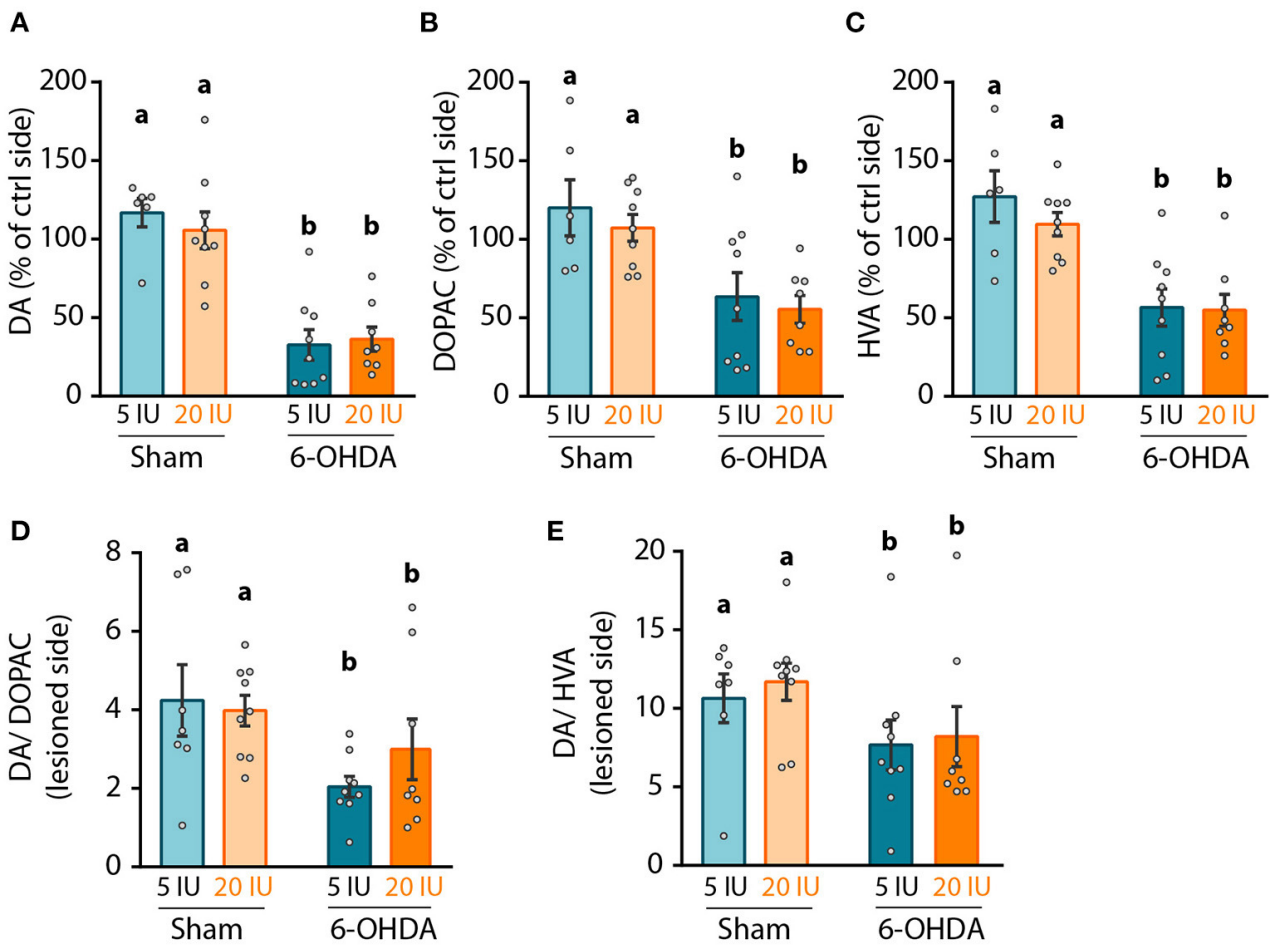
Impact of 6-OHDA lesion and dietary vitamin A on monoamines levels in the striatum. **(A–C)** Quantification of dopamine (DA) using HPLC coupled to electrochemical detection **(A)**, 3,4-Dihydroxyphenylacetic acid (DOPAC) **(B)**, and homovanillic acid (HVA) **(C)** in the striatum. Results are expressed in pmoles/ug of total proteins. **(D,E)** Expression of DA/DOPAC **(D)** and DA/HVA **(E)** ratios. Results are expressed as means ± SEM with individual data points. a, b: values significantly different (*p* < 0.005). Details of the statistical analysis are summarized in Table 3.

We then focused on the integrity of the DA system, in order to investigate the mechanisms by which vitamin A supplementation improved motor function. First, we measured the levels of DA and metabolites in striatal homogenates. Since no difference was observed in the contralateral side of the lesion between groups, results were normalized to the contralateral side. As expected, 6-OHDA lesion significantly reduced DA levels in the lesioned hemisphere (*p* < 0.001) compared to sham rats. Similar results were observed for two major degradation products of DA, namely 3,4-Dihydroxyphenylacetic acid (DOPAC) (*p* < 0.001) and homovanillic acid (HVA) (*p* < 0.001) (Figures 4A–C). Similarly, DA/DOPAC and DA/HVA ratios were significantly reduced by 6-OHDA lesion (*p* = 0.012 and *p* = 0.05 respectively) (Figures 4D,E), suggesting increased metabolism of DA. However, no effect of vitamin A supplementation was detected on monoamines measurements (Figures 4A–E).

**Figure 5:**
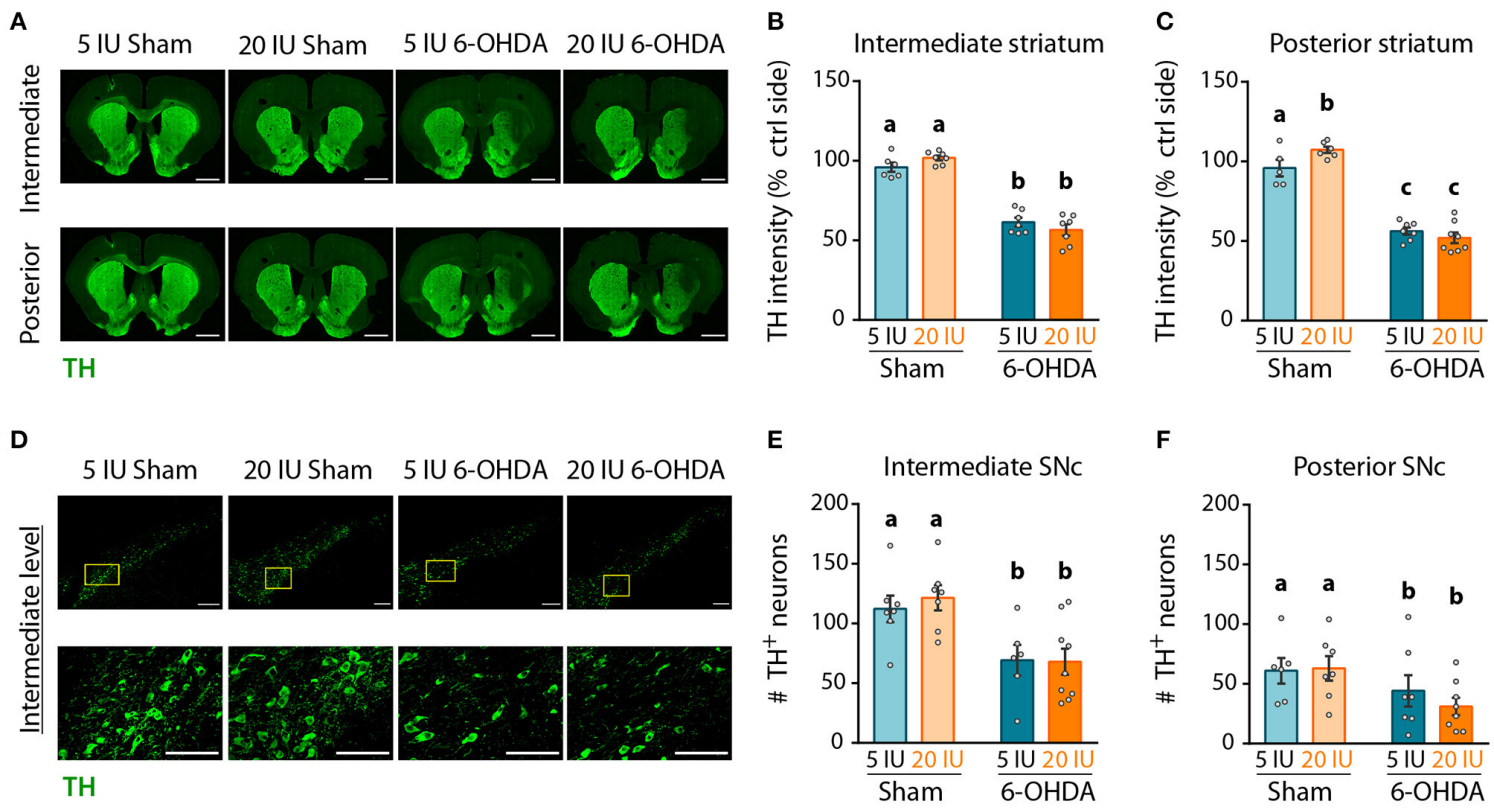
Impact of 6-OHDA lesion and dietary vitamin A on extent of DA fibers in the striatum. **(A)** Representative images for the 4 experimental groups of intermediate and posterior striatum (lesioned and control sides) immunostained for tyrosine hydroxylase (TH) (green). Scale bar: 1000 μm. **(B,C)** Quantification of fluorescence intensity of TH staining in intermediate **(B)** and posterior **(C)** striatum. TH intensity was normalized to the ipsilateral side. **(D)** Representative images for the 4 experimental groups of intermediate SNc (lesioned side) immunostained for TH (green). Top images correspond to a confocal mosaic of multiple images taken individually at 40X, scale bar: 200 μm. Bottom images correspond to a zoom from mosaic images (yellow squares in top images), scale bar: 100 μm. **(E,F)** Quantification of the number (#) of TH^+^ neurons in intermediate **(E)** and posterior **(F)** SNc. Results are expressed as means ± SEM with individual data points. a–c: values significantly different (*p* < 0.005). Details of the statistical analysis are summarized in Table 3.

Next, we quantified the intensity of the TH signal as a proxy for the density of DA fibers in the striatum by immunofluorescence, and the number of TH^+^ neurons in the SNc. Analyses were performed on three antero-posterior levels for the striatum and the SNc: anterior (bregma striatum: +2.2, SNC: −5.2); intermediate (bregma striatum: +1.6, SNC: −5.3); and posterior (bregma striatum: +0.7, SNC: −5.8). The anterior levels are presented in Supplementary Figures 2–4. In the striatum (Figure 5A, Supplementary Figures 2A,B), TH intensity was clearly reduced at the injection site, in 6-OHDA rats but not in sham rats, for both intermediate (5 IU sham vs. 5 IU 6-OHDA: *p* < 0.001; 20 IU sham vs. 20 IU 6-OHDA: *p* < 0.001) (Figure 5B), and posterior (5 IU sham vs. 5 IU 6-OHDA: *p* < 0.001; 20 IU sham vs. 20 IU 6-OHDA: *p* < 0.001) (Figure 5C) levels. This is in accordance with measurements of DA in the striatum (Figure 4). Of note, TH intensity in the posterior striatum was increased by vitamin A supplementation in sham rats, but not in 6-OHDA rats (5 IU sham vs 20 IU sham: *p* = 0.031). In the SNc (Figure 5D, Supplementary Figures 2A,C), the number of positive neurons significantly decreased in rats injected with 6-OHDA, in both intermediate (*p* < 0.001) (Figure 5E) and posterior (*p* = 0.028) (Figure 5F) levels, with no difference between dietary groups.

**Figure 6:**
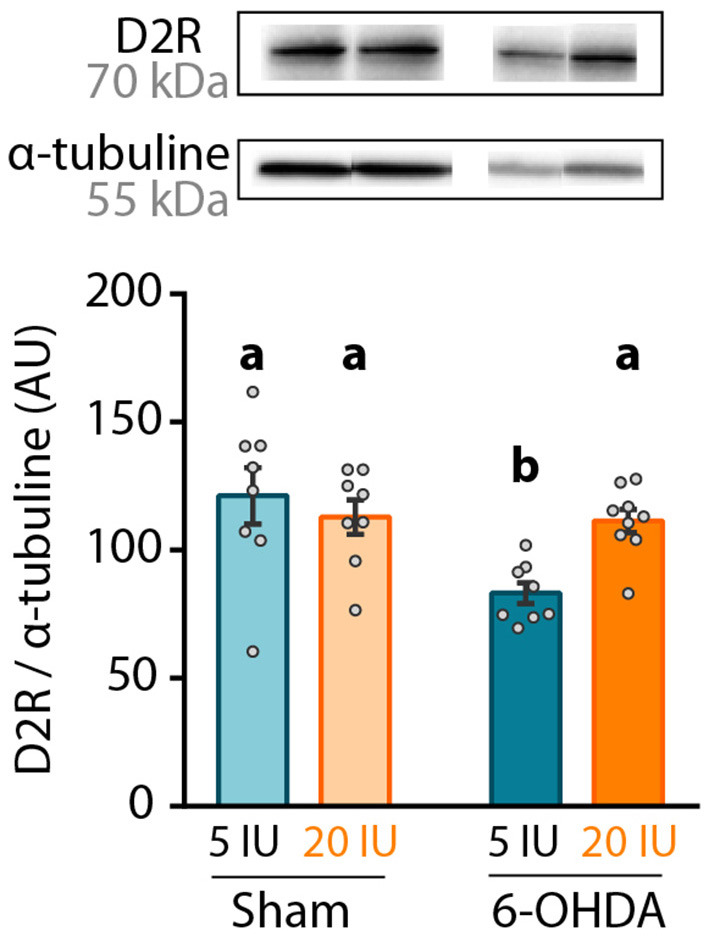
Impact of 6-OHDA lesion and dietary vitamin A on D2R protein levels in the striatum. Representative western blot for dopamine D2 receptor (D2R) in striatum homogenates (upper inset) and quantification (graph). Protein expression levels were normalized with α-tubuline for each sample. Results are expressed as means ± SEM with individual data points. a, b: values significantly different (*p* < 0.005). Details of the statistical analysis are summarized in Table 3.

Finally, dopamine D2 receptor (D2R) expression was measured in the striatum by western blot, since its expression is under the control of retinoic acid (32). We found that 6-OHDA injection significantly reduced D2R protein level in the striatum (Figure 6). However, this effect was not observed in 6-OHDA rats under supplemented diet, for which D2R expression levels were similar to sham rats (5 IU sham vs. 5 IU 6-OHDA: *p* < 0.001; 20 IU sham vs. 20 IU 6-OHDA: *p* = 0.882) (Figure 6). This suggests that increased retinoid signaling induced by vitamin A supplementation (Figure 2) prevented the decrease in striatal D2R expression in 6-OHDA rats. Of note, the decrease of D2R expression observed in 6-OHDA rats under sufficient diet is not usual, since studies have mainly found increased binding of D2R (33) or no change in D2R expression (34, 35). This discrepancy may be due to technical reasons, such as protein quantification vs. binding assays, or the conditions of the lesion.

### Impact of Vitamin A Supplementation on ALDH1A1^+^ Dopaminergic Neurons

**Figure 7:**
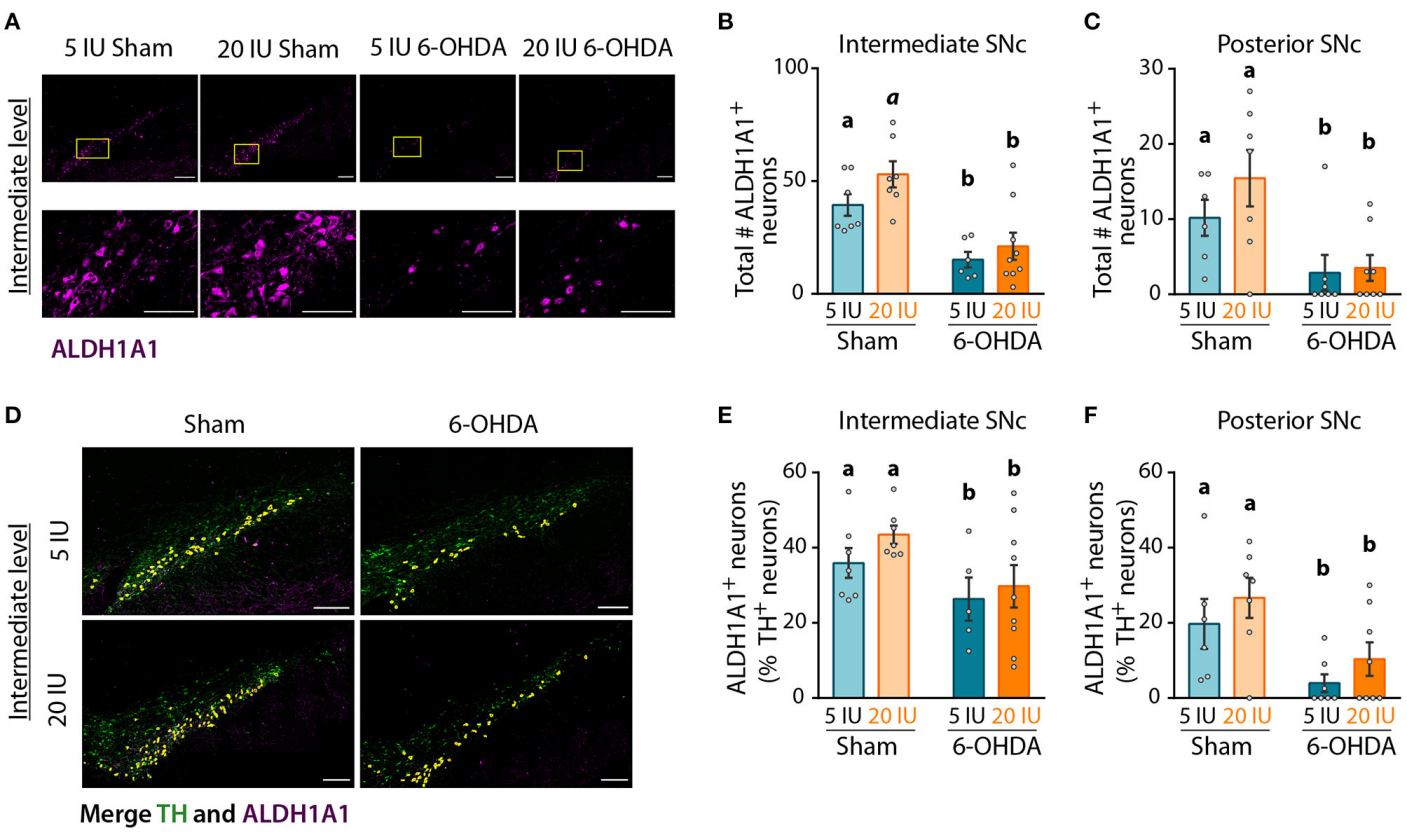
Impact of 6-OHDA lesion and dietary vitamin A on ALDH1A1^+^ DA neurons. **(A)** Representative images for the 4 experimental groups of intermediate SNc (lesioned side) immunostained for aldehyde dehydrogenase A subtype a1 (ALDH1A1) (magenta). Top images correspond to a mosaic of multiple images taken individually at 40X, scale bar: 200 μm. Bottom images correspond to a zoom from mosaic images (yellow squares in top images), scale bar: 100 μm. **(B,C)** Quantification of the number (#) of ALDH1A1^+^ neurons in intermediate **(B)** and posterior **(C)** SNc. **(D)** Representative images for the 4 experimental groups of intermediate SNc (lesioned side) immunostained for tyrosine hydroxylase (TH) (green) and ALDH1A1 (magenta). Co-staining is highlighted in yellow. Scale bar: 200 μm. **(E,F)** Quantification of the number (#) of ALDH1A1^+^ and TH^+^ neurons expressed in percent (%) of TH^+^ neurons in intermediate **(E)** and posterior **(F)** SNc. Results are expressed as means ± SEM with individual data points. a, b: values significantly different (*p* < 0.005); *a* indicate a trend for statistical significance (*p* = 0.09). Details of the statistical analysis are summarized in Table 3.

Analysis of global DA transmission in the nigro-striatal pathway did not reveal a clear effect of vitamin A supplementation, in comparison to motor improvement. This may be due to a specific impact of vitamin A on the sub-population of DA neurons expressing ALDH1A1. To test whether ALDH1A1^+^ DA neurons were specifically protected by vitamin A supplementation, we quantified the number of ALDH1A1^+^ DA neurons in the anterior, intermediate and posterior SNc (Figure 7A, Supplementary Figure 3). Analysis revealed that 6-OHDA lesion significantly decreased the number of ALDH1A1^+^ neurons in intermediate (*p* < 0.001) (Figure 7B) and posterior SNc (*p* = 0.001) (Figure 7C). Of note, we generally observed a tendency for more ALDH1A1^+^ neurons with vitamin A supplementation, in both sham and 6-OHDA rats. However, statistics only revealed a trend (*p* = 0.086) for the intermediate level of the SNc (Figure 7B).

Since ALDH1A1^+^ neurons are also TH^+^ neurons, we quantified the proportion of TH^+^ neurons that expressed ALDH1A1 (Figure 7D), in order to identify this sub-population of DA neurons. In sham rats fed with sufficient diet, the proportion of TH^+^ neurons that expressed ALDH1A1 was 36, 36, and 20%, for the anterior, intermediate and posterior levels of the SNc, respectively. These proportions are smaller than previous reports in the literature, with about 70% in mice as in humans (15, 16, 18). This difference may be due to the rat model, as well as the analysis methods. Proportions of ALDH1A1^+^ DA neurons were significantly reduced by 6-OHDA lesion, at every level of the SNc (Figures 7E,F, Supplementary Figure 3) (8E: *p* = 0.025; 8F: *p* = 0.002). Of note, a trend for a higher proportion of TH^+^ neurons expressing ALDH1A1 was observed in vitamin A supplemented rats.

**Figure 8:**
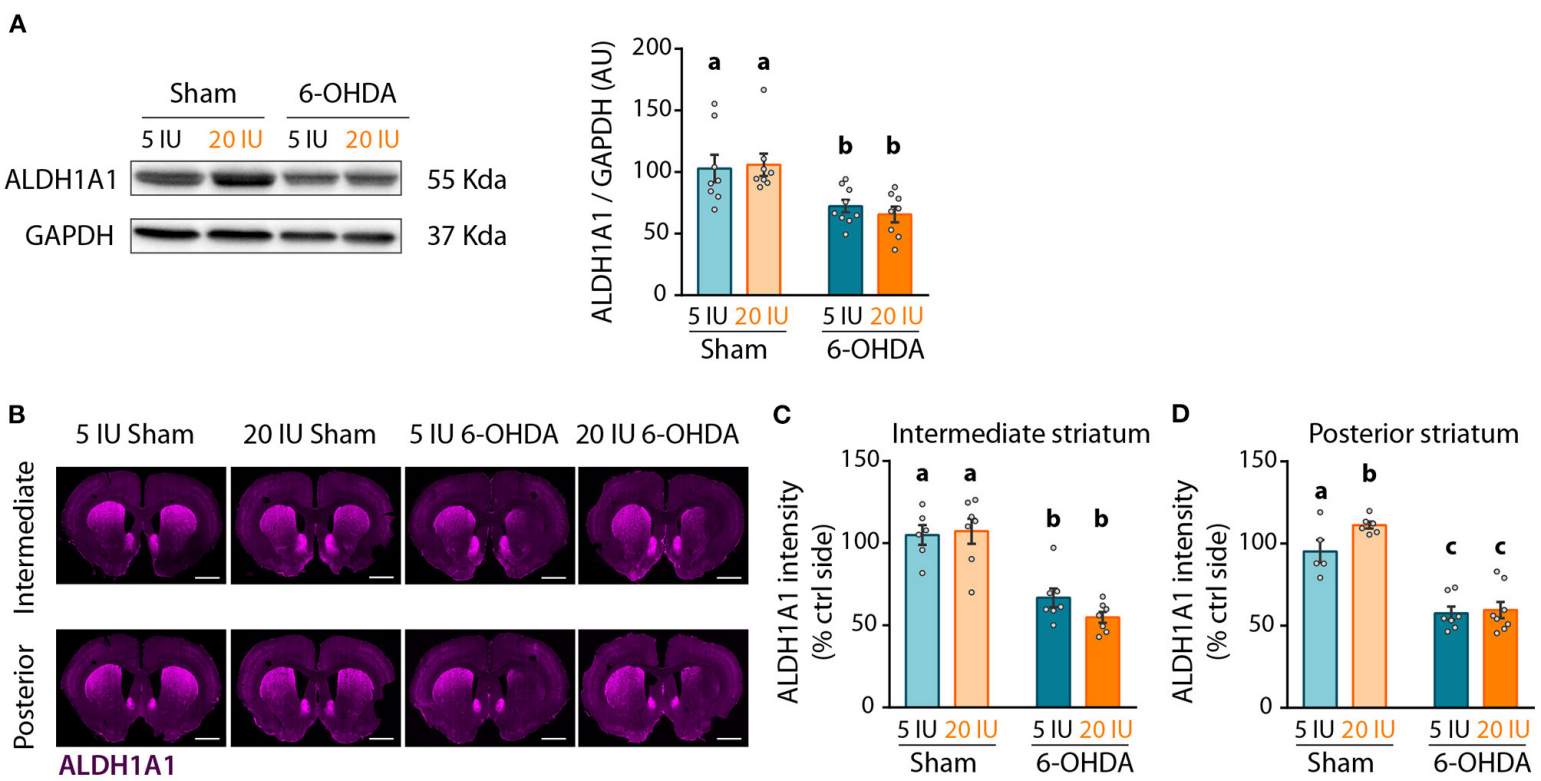
Impact of 6-OHDA lesion and dietary vitamin A on extent of ALDH1A1^+^ fibers in the striatum. **(A)** Representative western blot for aldehyde dehydrogenase A subtype a1 (ALDH1A1) in striatum homogenates (left inset) and quantification (graph). Protein expression levels were normalized with GAPDH for each sample. **(B)** Representative images for the 4 experimental groups of intermediate and posterior striatum (lesioned and control sides) immunostained for ALDH1A1 (magenta). Scale bar: 1000 μm. **(C,D)** Quantification of fluorescence intensity of ALDH1A1 staining in intermediate **(C)** and posterior **(D)** striatum. ALDH1A1 intensity is normalized to the ipsilateral side. Results are expressed as means ± SEM with individual data points. a–c: values significantly different (*p* < 0.005). Details of the statistical analysis are summarized in Table 3.

Lastly, we quantified ALDH1A1 expression in the striatum by western-blot, as a reflect of the amount of DA afferents from ALDH1A1^+^ DA neurons. As for ALDH1A1^+^ neurons in SNc, 6-OHDA injection significantly reduced ALDH1A1 levels in the striatum compared to sham-injected rats (*p* < 0.001) (Figure 8A). However, no effect of vitamin A supplementation was observed.

We refined the analysis with ALDH1A1 staining, in the anterior, intermediate and posterior parts of the striatum (Figure 8B, Supplementary Figure 4). Confirming western blot measurements, 6-OHDA lesion significantly reduced ALDH1A1 staining in the striatum, at the anterior (*p* = 0.033, Supplementary Figure 4B), intermediate (*p* < 0.001, Figure 8C) and posterior (*p* < 0.001, Figure 8D) levels. Vitamin A supplementation induced no effect on ALDH1A1 staining in the striatum of 6-OHDA rats. By contrast, sham rats supplemented with vitamin A showed increased ALDH1A1 staining in the posterior striatum, compared to sham rats fed sufficient diet (5 IU sham vs. 20 IU sham: *p* = 0.039).

## Discussion

In this study, we examined the impact of preventive vitamin A supplementation in a PD rat model on motor behavior and dopamine transmission with the initial hypothesis that vitamin A would protect ALDH1A1^+^ DA neurons.

First of all, when one is studying vitamin A supplementation, the levels of vitamin A intake have to be precisely controlled since chronic high doses of vitamin A can lead to oxidative stress and toxicity for the liver (8, 36, 37). In rats, common levels for sufficient retinol intakes range between 5 and 8 IU/g of diet, while vitamin A supplementation ranges from 20 to ~50 IU/g (31, 38, 39). In the current study, we used a dose at the bottom range of supplementation, with 20 IU/g of diet, which corresponds to ~600 IU/ day or 180μg retinol/day. This level of vitamin A supplementation is considered as comparable to therapeutic doses used in humans (40, 41). However, the toxicity of this dose is unclear. Indeed, oxidative stress has been observed (40, 41), but other reports showed no deleterious effect on behavior and inflammatory status (31, 38). In our conditions, vitamin A supplementation (20 IU/g of diet) for 8 weeks was sufficient to increase retinol in the liver, the storage organ for retinol, but we did not identify any adverse effect of vitamin A supplementation, as illustrated by measures of body weight, motor function and DA transmission. Thus, we conclude that in our conditions, vitamin A supplementation was adequate.

Second, we explored the impact of 6-OHDA lesion and vitamin A supplementation on motor function using 3 complementary motor tests (23, 42). We did not observe strong effect of 6-OHDA and vitamin A supplementation in the rotarod test, and only an effect of the injection in the step test. In both tests, the animal was forced to move to maintain its equilibrium. By contrast, our data revealed positive effects of vitamin A supplementation in the cylinder test, where the animals were free to make voluntary movements, in accordance with their natural instinct of exploration (43). Voluntary movements are controlled by the implicit motivation of producing movement, which is modulated by nigrostriatal DA neurons (16, 44). Yet, motor motivation is reduced in PD patients, as measured by reduced vigor of movement (45, 46). Here, our data suggests that vitamin A supplementation in 6-OHDA lesioned rats has a beneficial impact on motor function through the improvement of motivation for voluntary movements.

In order to identify neurobiological mechanisms by which vitamin A supplementation improved motor function in 6-OHDA lesion rats, we focused our analyses on ALDH1A1 expressing DA neurons. These neurons constitute the sub-population of DA neurons that are preferentially degenerating in PD patients (15, 16), and their role in motor learning and motor vigor has been recently revealed in mice (18). Intriguingly, these neurons lose their ability to express ALDH1A1 before degenerating, suggesting that ALDH1A1 enzyme may have a protective effect for these neurons (15, 47). Yet, ALDH1A1 expression is controlled by retinoic acid, the active metabolite of vitamin A, therefore, we initially hypothesized that vitamin A intake was able to modulate ALDH1A1 expression in SNc neurons.

In humans, ALDH1A1 is also an enzyme that raises interest, since it has been identified as a potential biomarker for PD (15, 48–50). Indeed, mRNA levels of ALDH1A1 in peripheral blood are significantly decreased in PD patients compare to control cases (50). Moreover, clinical studies showed that the genetic variability of *ALDH1A1* is a good predictive factor for PD diagnosis and the progress rate of the disease (51–53). Yet, if vitamin A is necessary for ALDH1A1 expression, a decreased vitamin A bioavailability may constitute a risk factor for the disease. However, no clear link has been established yet in PD patients between vitamin A function, ALDH1A1 expression and PD symptoms (8).

Here, we show that in sham rats, vitamin A supplementation tended to increase the number of ALDH1A1^+^ DA neurons in the SNc, which leads to a significant increase of ALDH1A1 fibers in the posterior striatum. This supports the fact that dietary vitamin A supplementation can sustain ALDH1A1 expression in DA neurons. However, this increase of ALDH1A1^+^ neurons and fibers in the striatum with vitamin A supplementation was not significant in 6-OHDA rats. This may be explained by the delay after the lesion chosen here. Indeed, it is possible that protection of ALDH1A1^+^ neurons with vitamin A supplementation was effective after the lesion but not maintained in time. In addition, we used a mild supplementation (20 IU/g of diet), therefore, a higher vitamin A intake, still in therapeutic range (e.g. 40 IU/g of diet) may exhibit more pronounced effects on 6-OHDA-induced motor and neurobiological alterations. Furthermore, ALDH1A1^+^ DA neurons are distributed in certain territories of the SNc and ALDH1A1^+^ fibers are preferentially innervating striosomes compartments of the striatum (16, 54–56). Therefore, a more detailed analysis of ALDH1A1^+^ DA neurons, accounting for these sub-territories may reveal a more specific impact of vitamin A on ALDH1A1^+^ neurons.

Finally, this work revealed that the stronger improvement induced by vitamin A supplementation in 6-OHDA rats in addition to voluntary movement, concerned RXRγ and D2R expression in the striatum. RXRγ is a sub-type of RXR; its expression is directly controlled by retinoic acid levels and its most potent endogenous ligand is 9-cis-dihydro retinoic acid (57). The striatum is the brain structure containing the highest levels of RXRγ (58), indicating its crucial role for striatum function. Through dimerization with other nuclear receptors, such as RARβ or Nur77, RXRγ has an important function for the development of the striatum and the nigro-striatal pathway (59–61), and for dopamine transmission at adult age (62). As a consequence, mice lacking RARβ and/or RXRγ exhibit strong motor impairments along with alteration of DA transmission, analog to PD mice models (58). Of note, RXRγ controls the expression of D2R in the striatum (32, 63). Here, our data shows that increased retinol level in the liver is reflected in the striatum by the increased expression of RXRγ. Consequently, D2R expression is also significantly increased in 6-OHDA rats under vitamin A supplementation, compared to 6-OHDA rats under sufficient diet. This increase in D2R expression may explain improved motor function in 6-OHDA rats under vitamin A supplementation. For future studies, a deeper analysis of retinoid and dopamine receptors may help to better understand the role of retinoid signaling in improving striatal function with vitamin A supplementation.

In conclusion, our study revealed beneficial impact of vitamin A supplementation on striatal function, with improved voluntary movements, increased expression of RXRγ and D2R in the striatum, and a trend for more ALDH1A1^+^ DA neurons and fibers. A more sustained supplementation (amount and/or duration) with a more degenerative rat model, such as expression of human alpha-synuclein in SNc (64) may help to better understand the role of dietary vitamin A in the survival of DA neurons in the context of PD. In addition, this work may trigger future research to investigate the beneficial effect of preventive vitamin A supplementation in patients, particularly for those with low vitamin A metabolism.

## Data Availability Statement

The raw data supporting the conclusions of this article will be made available by the authors, without undue reservation.

## Ethics Statement

The animal study was reviewed and approved by Comité d'éthique de Bordeaux - CEEA 50.

## Author Contributions

CB-B: conceptualization and project administration. CB-B and SV: funding and supervision of staff. AM, JL, MD, SA, VS-P, ER, SV, and CB-B: experiments. AM and CB-B: statistical analyses and writing—original draft. AM: visualization. FD, RG, CB, AM, SA, CB-B, and VP: writing—review and editing. All authors contributed to the article and approved the submitted version.

## Funding

This work was supported by France Parkinson. PhD fellowship for AM was provided by French Ministry. The Bordeaux Imaging Center is supported by the French National Research Agency (ANR-10-INBS-04). The Bioprot Platform of the Neurocampus facilities at the University of Bordeaux is funded by the LABEX BRAIN (ANR-10-LABX-43).

## Conflict of Interest

The authors declare that the research was conducted in the absence of any commercial or financial relationships that could be construed as a potential conflict of interest.

## Publisher's Note

All claims expressed in this article are solely those of the authors and do not necessarily represent those of their affiliated organizations, or those of the publisher, the editors and the reviewers. Any product that may be evaluated in this article, or claim that may be made by its manufacturer, is not guaranteed or endorsed by the publisher.

## Abbreviations

6-OHDA: 6-hydroxydopamine
TH: tyrosine hydroxylase
ALDH1A1: aldehyde dehydrogenase 1 subtype A1
SNc: substantia nigra *pars compacta*
PD: Parkinson's disease
DA: dopamine
RXR: retinoid X receptor
RAR: retinoic acid receptor
D2R: dopamine D2 receptor
IBA1: Ionized calcium binding adapter molecule 1
DOPAC: dihydroxyphenylacetic acid
HVA: homovanillic acid
GAPDH: glyceraldehyde 3-phosphate dehydrogenase
HPLC: high performance liquid chromatography
Ctrl: control
PBS: phosphate buffer saline.
