# Supplementary material for: Preventive Vitamin A Supplementation Improves Striatal Function in 6-Hydroxydopamine Hemiparkinsonian Rats
Source: Front Nutr. 2022 Feb 1;9:811843. doi: 10.3389/fnut.2022.811843 (PMC8843942; doi:10.3389/fnut.2022.811843)
Supplement: Supplementary file 1 [file Data_Sheet_1.docx]

**SUPPLEMENTAL MATERIALS**

**Supp. Fig. 1. Impact of 6-OHDA lesion and dietary vitamin A on activated microglia in the striatum**

(**A**) Representative immunofluorescence images of intermediate striatum labelled with Ionized calcium binding adapter molecule 1 (IBA1) (green) for the 4 experimental groups. The square shows a zoom on IBA1⁺ cells in the striatum. Scale bar: 1000 µm for whole striatum and 25 µm for zoom images. (**B, C**) Quantification of density of IBA1⁺ cells/area (mm²) in intermediate striatum (B) and posterior striatum (C). (**D, E**) Quantification of fluorescence intensity of IBA1 staining in intermediate striatum (D) and posterior striatum (E). The fluorescence intensity is ranging from 0 to 255. Results are expressed as means ± SEM with individual data points. No significant difference was found. Details of the statistical analysis are summarised in (**Supp.** **Table. 1**).

**Supp. Fig. 2. Extent on DA fibres in the anterior striatum and DA neurons in the anterior SNc**

(A) Representative immunofluorescence images, for the 4 experimental groups, of anterior striatum (lesioned side on the right) and SNc (lesioned side only) immunostained for tyrosine hydroxylase (TH) (green). The scale bar corresponds to 1000 µm for striatum images and 200 µm for SNc images. (B) Quantification of fluorescence intensity of TH staining in anterior striatum. TH intensity is expressed as percentage (%) of the ipsilateral side. (C) Quantification of number (#) of TH⁺ neurons in anterior SNc. Results are expressed as means ± SEM with individual data points for histogram. a-b: values significantly different. Details of the statistical analysis are summarized in (**Supp.** **Table. 1**).

**Supp. Fig. 3. Immunostaining of ALDH1A1 positive dopaminergic neurons in anterior SNc**

(A) Representative immunofluorescence images, for the 4 experimental groups, of anterior SNc (lesioned side) immunostained tyrosine hydroxylase (TH) (green) and aldehyde dehydrogenase A subtype a1 (ALDH1A1) (magenta). Co-staining is highlighted in yellow. Scale bar: 200 µm. (B) Quantification of number (#) of ALDH1A1⁺ neurons in anterior SNc. (C) Quantification of number of ALDH1A1⁺ and TH⁺ neurons expressed in percent (%) of TH⁺ neurons in anterior SNc. Results are expressed as means ± SEM with individual data points for histogram. No significant differences have been found. Details of the statistical analysis are summarized in (**Supp.** **Table. 1**).

**Supp. Fig. 4. Immunostaining of ALD1A1 in the anterior striatum**

(A) Representative immunofluorescence images, for the 4 experimental groups, of anterior striatum (lesioned and control side) immunostained for ALDH1A1 (magenta). The scale bar corresponds to 1000 µm. (B) Quantification of fluorescence intensity of ALDH1A1 staining in anterior striatum. ALDH1A1 intensity is expressed as percentage (%) of the ipsilateral side. Results are expressed as means ± SEM with individual data points for histogram. a-b: values significantly different. Details of the statistical analysis are summarized in (**Supp.** **Table. 1**).

**Supp. Table 1. Summary of statistical analysis**

Statistical significance was assessed as #p < 0.09, *p < 0.05, **p < 0.01, ***p < 0.001.
