# Supplementary figures and images for: Preventive Vitamin A Supplementation Improves Striatal Function in 6-Hydroxydopamine Hemiparkinsonian Rats
Source: Front Nutr. 2022 Feb 1;9:811843. doi: 10.3389/fnut.2022.811843 (PMC8843942; doi:10.3389/fnut.2022.811843)

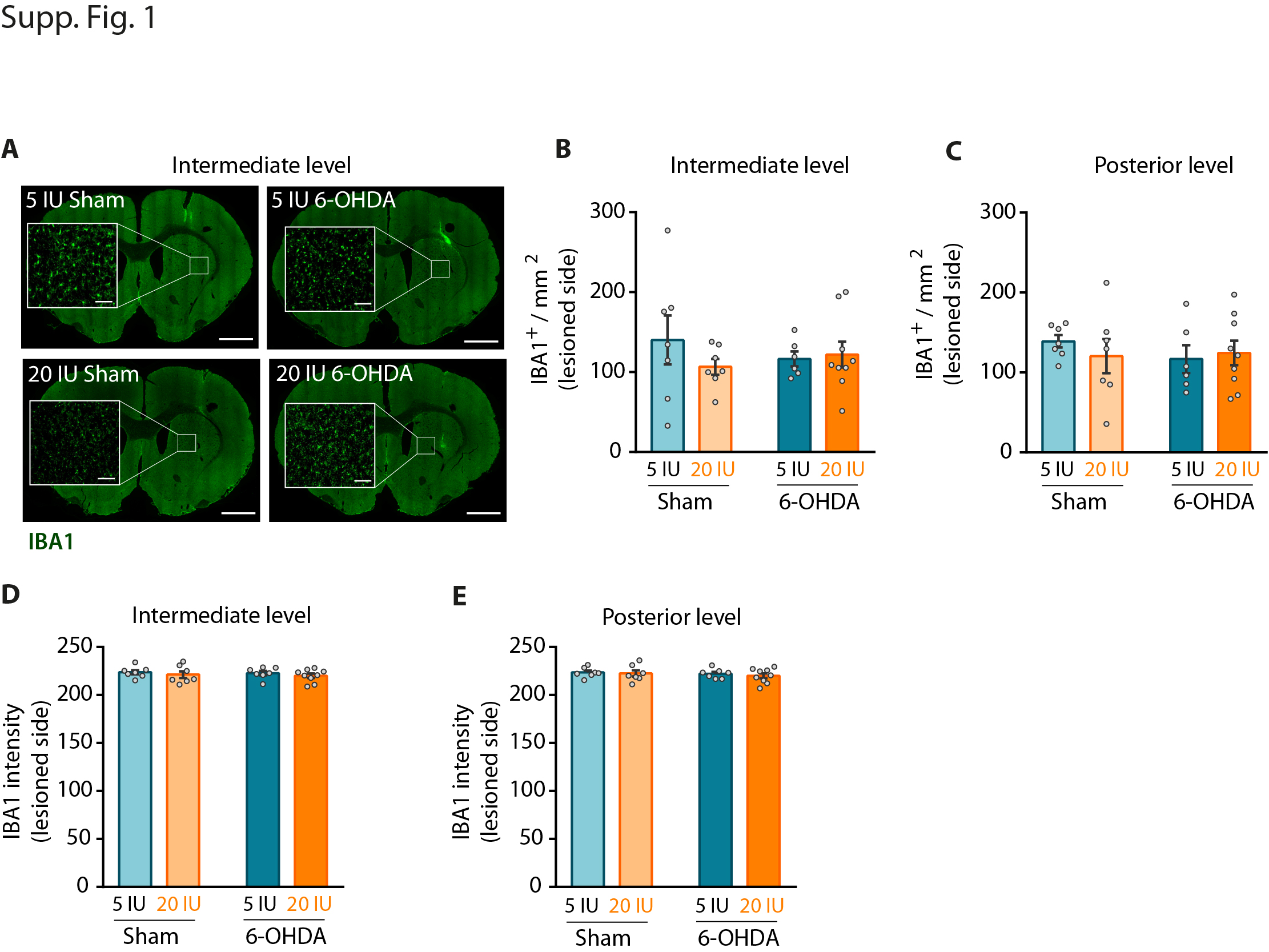

Supplement: Supplementary file 2 [file Image_1.JPEG]

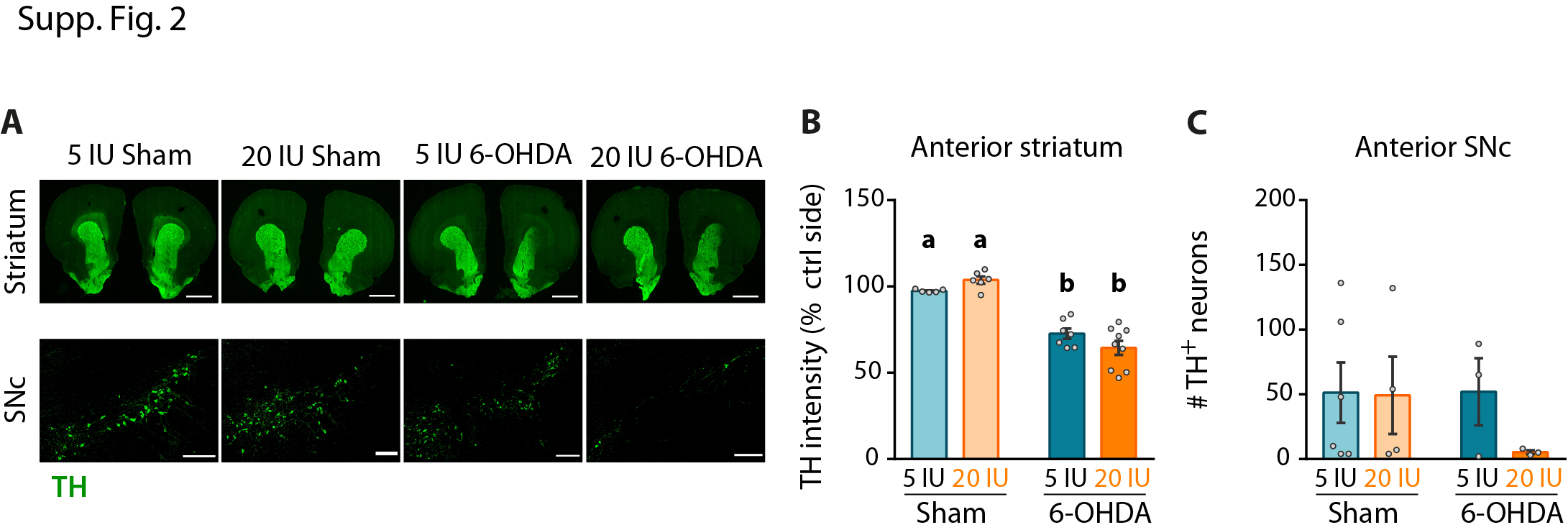

Supplement: Supplementary file 3 [file Image_2.JPEG]

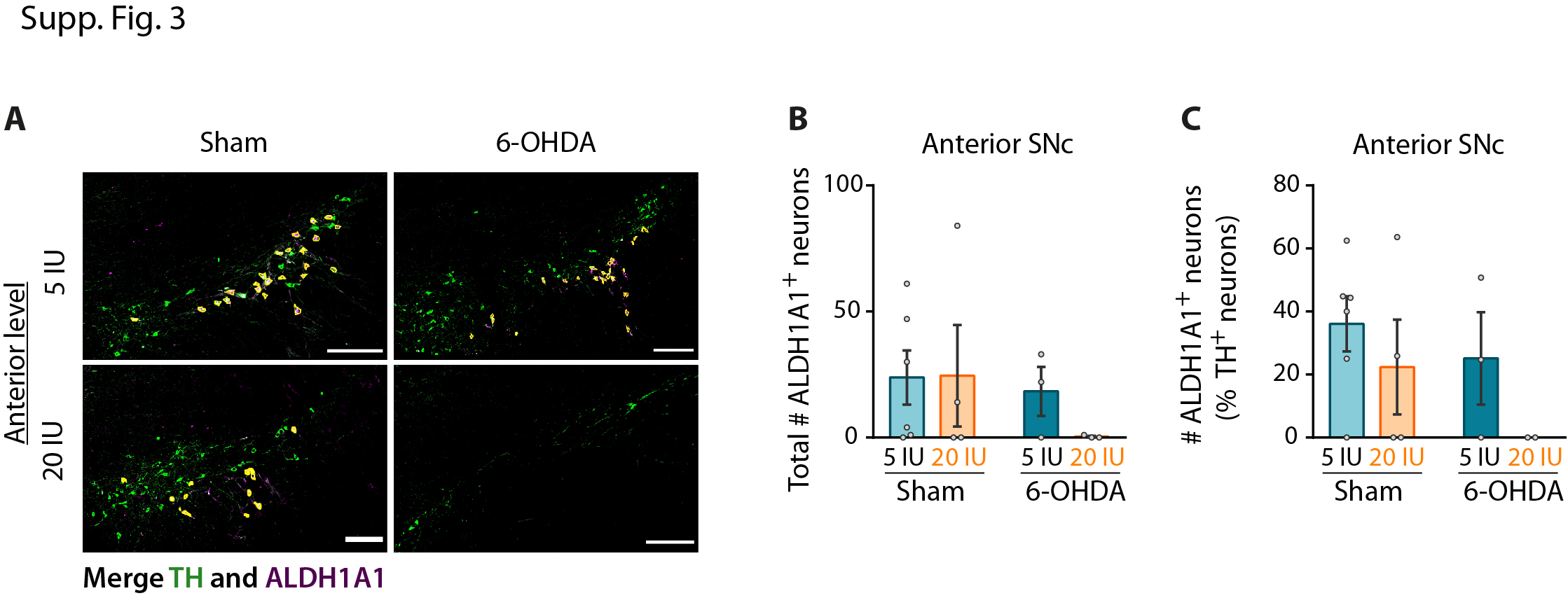

Supplement: Supplementary file 4 [file Image_3.JPEG]

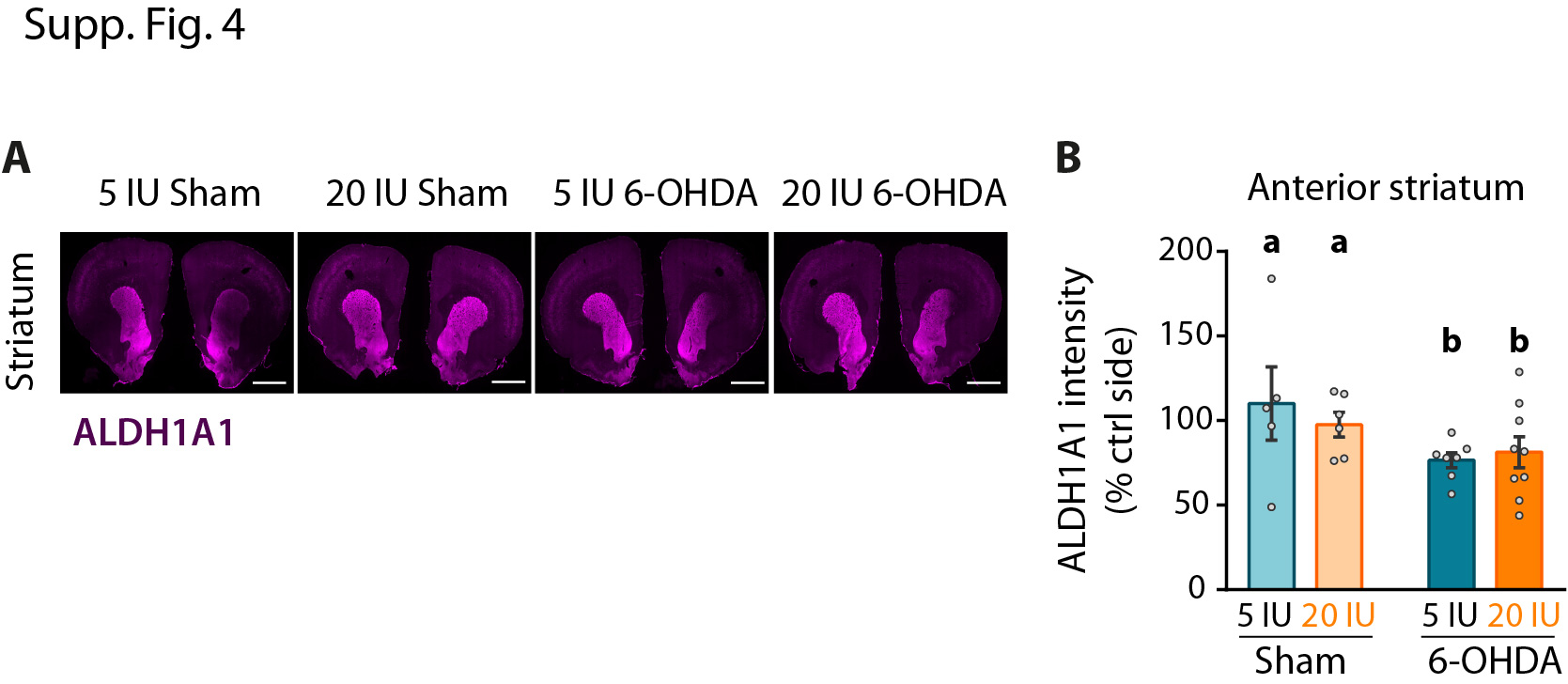

Supplement: Supplementary file 5 [file Image_4.JPEG]
